# Comparing the Effects of General Versus Regional Anesthesia on Postoperative Mortality in Total and Partial Hip Arthroplasty

**DOI:** 10.7759/cureus.12462

**Published:** 2021-01-03

**Authors:** Irfan A Khan, Raihan Noman, Nabeel Markatia, Grettel Castro, Pura Rodriguez de la Vega, Juan Ruiz-Pelaez

**Affiliations:** 1 Department of Translational Medicine, Florida International University Herbert Wertheim College of Medicine, Miami, USA

**Keywords:** partial hip arthroplasty, general anesthesia, total hip arthroplasty, regional anesthesia, postoperative mortality

## Abstract

Purpose

Total hip arthroplasty (THA) and partial hip arthroplasty (PHA) are performed in patients with hip joint dysfunction such as osteoarthritis or hip fractures and are associated with complications including mortality. There is a lack of evidence in the literature regarding whether the type of anesthesia (regional vs. general) is associated with increased postoperative mortality in patients undergoing hip arthroplasty. The present study compares early postoperative mortality between general or regional anesthesia administered to patients undergoing either THA or PHA.

Methods

A retrospective cohort was assembled using the 2015-2016 American College of Surgeons National Surgical Quality Improvement Program database. Adult patients undergoing hip arthroplasty under general or regional anesthesia were included. Patients were excluded if receiving any other type of anesthesia, as well as having an American Society of Anesthesiologists (ASA) physical status classification score ≥ 4, preoperative acute renal failure, severe congestive heart failure (CHF), chronic obstructive pulmonary disease (COPD), or ascites. Adjusted odds of 30 days all-cause postoperative mortality according to the type of anesthesia were estimated by fitting multiple logistic regression models that included potential confounders and effect modifiers.

Results

A total of 60,897 patients were included in the study. Given that the interaction between the type of anesthesia and the type of arthroplasty was statistically significant, separated models were fitted for each type of arthroplasty. There was no evidence of an association between type of anesthesia and postoperative mortality in hip arthroplasty patients regardless of whether the arthroplasty was partial (odds ratio {OR} = 0.85; confidence interval {CI} 0.59-1.22) or total (OR = 0.68; CI 0.43-1.08).

Conclusion

The overall early postoperative mortality in adult hip arthroplasty patients is low in the absence of risk factors such as severe CHF, COPD, ascites, acute renal failure, and ASA score of 4 or higher. Our findings suggest there is no association between the type of anesthesia received (general vs. regional) and early postoperative mortality rates in patients undergoing hip arthroplasty, regardless of type (total vs. partial).

## Introduction

Hip arthroplasty is a surgical procedure that replaces the damaged part(s) of the hip joint with artificial parts called prostheses [1]. It is commonly performed in patients to restore functional mobility of the hip joint and reduce pain. Indications for surgery include hip osteoarthritis, femoral neck fractures, avascular necrosis of the femoral head, infection, and others [2-5]. Total hip arthroplasty (THA) is regularly performed for patients with hip osteoarthritis, and it consists of replacing both bony structures of the hip joint, the femoral head and the acetabulum, with prostheses. Partial hip arthroplasty (PHA) is indicated in some patients with femoral neck fractures, and it consists of replacing only the damaged portion of the hip joint, the femoral head, with a prosthesis [6]. THA and PHA are commonly performed procedures, with THAs having a particularly high volume of over 370,000 performed in 2014 in the United States, and a projected 635,000 to be performed in 2030 [7]. THAs have proven to be particularly effective for revision in the treatment of hip dislocation and mechanical joint loosening [8]. However, hip arthroplasty is not without its consequences. Perioperative complications include death, pulmonary embolism, thromboembolism, sepsis, and fractures [9].

In an effort to identify risk factors for adverse effects, many studies have evaluated the use of anesthesia and its impact on perioperative complications of hip arthroplasty. The choice of anesthesia type in hip arthroplasty is commonly debated by anesthesiologists and surgeons, particularly when choosing between regional and general anesthesia [10]. In regional anesthesia, patients remain responsive, but the targeted area is marked by loss of sensation. Subtypes of regional anesthesia include spinal, epidural, nerve blocks, etc. In general anesthesia, patients are in a complete state of unconsciousness [11]. The type of anesthesia utilized in hip arthroplasty patients is worth exploring further as it has important implications for the frequency and severity of perioperative complications given the differences in the mechanism of action and the rapidly increasing prevalence of hip arthroplasty [12]. Choice of anesthesia is important as well for primary care providers and cardiologists completing preoperative clearance for patients undergoing THA or PHA. 

Across several studies on this topic, the definition of regional anesthesia was not uniform, with some studies evaluating spinal anesthesia and others exploring epidural anesthesia, both of which are subtypes of regional anesthesia [12-15]. In addition, there have been conflicting results reported on the perioperative complications associated with regional anesthesia. One study found that regional anesthesia was associated with significantly higher odds of minor and total perioperative complications [16]. However, another study found that regional anesthesia significantly reduced deep surgical site infections as well as cardiovascular and pulmonary complications [17]. Moreover, only three studies analyzed the effect of regional versus general anesthesia on postoperative mortality, and none of them yielded a significant association [13,15,18]. One meta-analysis had a small sample size with a low prevalence of mortality [18], while the other meta-analysis included studies that also examined total knee replacements [14]. Basques et al. are the most similar to our study, but its sample size was much smaller and it only examined total hip arthroplasty [13].

Currently, it remains unclear if there is an association between the type of anesthesia used in total or partial hip arthroplasty patients and early postoperative mortality. Additionally, no studies have evaluated this association in partial hip arthroplasty patients. Since patients who receive PHA's may present with more emergent conditions when compared to patients undergoing THA's, it is important to investigate the association of the type of anesthesia used and postoperative mortality in these patients separately. The objective of our study is to answer the following: in adults at least 18 years of age undergoing total or partial hip arthroplasty, is the use of regional anesthesia associated with a lower early postoperative mortality rate (within 30 days) when compared to that of general anesthesia? We hypothesize that the use of regional is not associated with lower mortality in THA or PHA patients when compared with general anesthesia.

## Materials and methods

Research design

A retrospective cohort study was performed using data from the 2015-2016 American College of Surgeons National Surgical Quality Improvement Program (ACS NSQIP) database.

Data source

The ACS NSQIP database is compiled from hospitals across the nation, which report perioperative complications for 30 days following surgery as well as a multitude of variables, including various surgical parameters. Data quality in the database is high, as more than 95% of patients have 30-day follow-up data, and validity and accuracy checks are consistently performed.

Study population and sample

Participants in this study were patients at least 18 years of age in the United States, who underwent hip arthroplasty and received either general anesthesia or regional anesthesia. Patients were excluded if they received any other type of anesthesia, as well as those with ASA, score ≥ 4 (4 = severe systemic disease that is a constant threat to life; 5 = moribund; 6 = declared brain-dead). Patients who had any of the following conditions in the 30 days prior to surgery were also excluded: acute renal failure, severe congestive heart failure, COPD, or ascites.

Variables

The outcome of interest was all-cause postoperative mortality within 30 days of surgery. The exposure variable was defined as the type of anesthesia patients were given during the hip arthroplasty: general anesthesia or regional anesthesia (which includes regional, epidural, and spinal anesthesia according to the ACS NSQIP database). Covariates assessed were: type of arthroplasty (THA = total hip arthroplasty and PHA = partial hip arthroplasty), age, gender, body mass index (BMI), current smoking status within one year, diabetes mellitus with use of oral agents or insulin, hypertension requiring medication, disseminated cancer, steroid use for a chronic condition, functional health status prior to surgery, elective surgery, total operation time, and ASA classification.

Statistical analysis

An exploratory analysis was performed to profile the included sample, check data completeness and quality, and transform variables as needed. Two bivariate analyses were performed: (i) a comparison of the distribution of all control variables according to the exposure and (ii) a frequency of the outcome according to all independent variables, including the type of anesthesia. After testing for collinearity, a binary unconditional multiple logistic regression model was fitted, including potential confounders and a first-order interaction term between the type of hip arthroplasty (THA or PHA) and the type of anesthesia (regional or general), to estimate the adjusted odds ratio and 95% confidence interval of 30 days postoperative mortality and type of anesthesia. In case the interaction term was found to be statistically significant, provisions were made to fit two separate logistic regression models: one for patients undergoing partial hip arthroplasty and one for patients undergoing total hip arthroplasty.

Ethical aspects

The exemption was granted by the Florida International University IRB, given that this study was classified as non-human subjects research since it was a secondary data analysis from the ACS NSQIP database, a fully de-identified national database that protects patient information under HIPAA and the ACS NSQIP hospital participation agreement.

## Results

**Figure 1:**
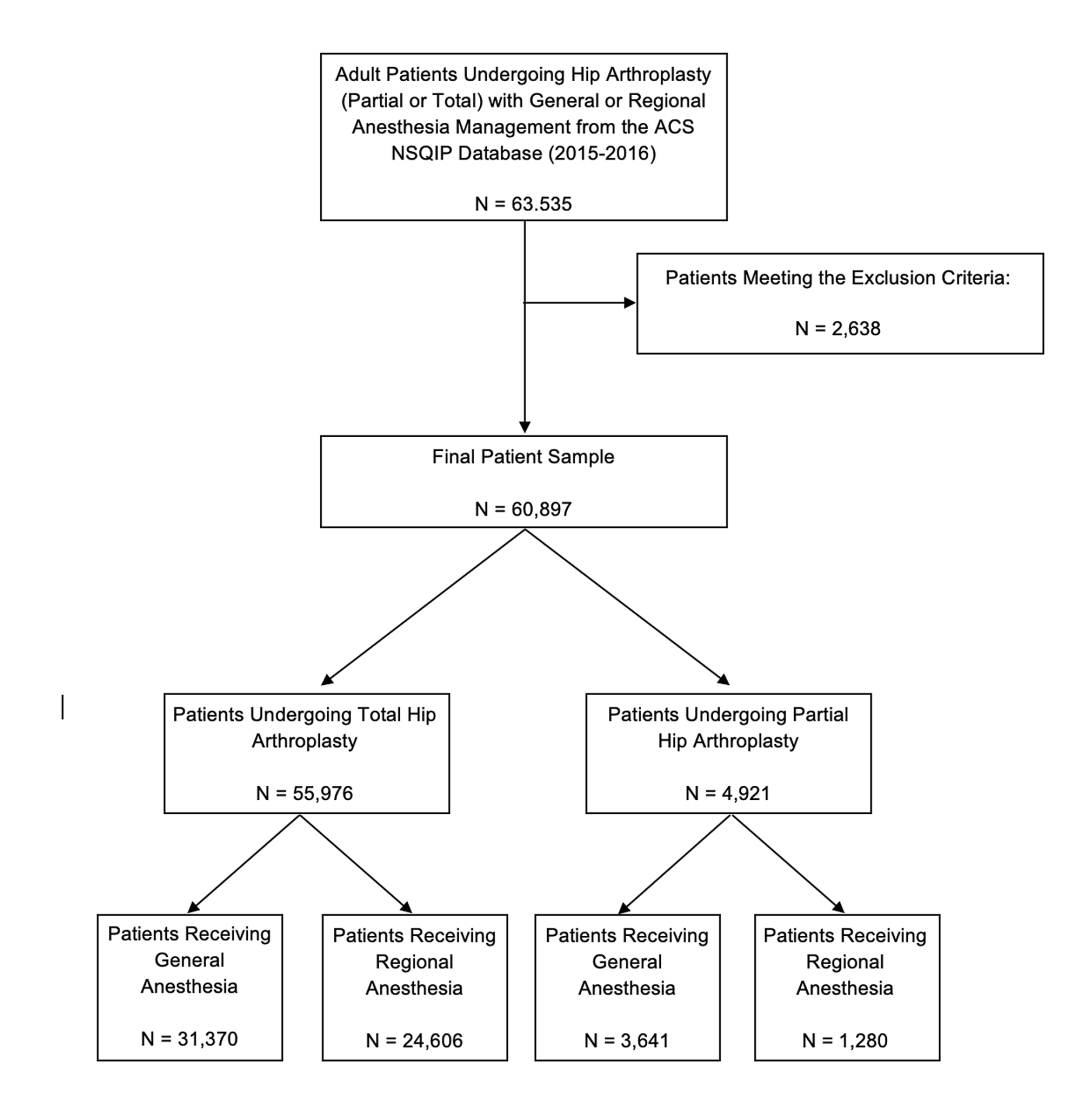
Selection Criteria for Sample Population ACS NSQIP: American College of Surgeons National Surgical Quality Improvement Program

There were 63,535 individuals in the ACS NSQIP database (2015-2016) who were initially eligible for our study (Figure 1). After excluding patients with an ASA classification of 4 or greater (N = 2638), the final effective sample size was 60,897 with 35,011 patients in the general anesthesia group and 25,886 patients in the regional anesthesia group. In both groups, most patients were female, and within the age group of 65-74 years old.

Differences in baseline characteristics between patients who received general anesthesia or regional anesthesia are displayed in Table 1. There were statistically significant differences (p < 0.05) between the two groups for all of the baseline characteristics and potential confounders. The most clinically significant differences involved age, BMI, elective surgery status, and ASA classification. Patients who received general anesthesia were more frequently in the 65-74 years old age group, and more frequently had elective surgery. Also, patients who received general anesthesia were more likely to be of the Class III obesity and the ‘Severe Systemic Disease’ ASA classifications.

**Table 1:** Characteristics of Patients Undergoing Hip Arthroplasty With General Anesthesia vs. Regional Anesthesia (N = 60,897) ^a^“Regional anesthesia” is defined as patients who received intraoperative regional, epidural, or spinal anesthesia, as per the ACS NSQIP database. ^b^“No/mild disturb” is defined as patients under the ASA classification (1) no disturb or (2) mild disturb. BMI: body mass index; ACS NSQIP: American College of Surgeons National Surgical Quality Improvement Program; ASA: American Society of Anesthesiologists

Patient Characteristics	Type of Intraoperative Anesthesia				P-Value
	General Anesthesia (35,011)		Regional Anesthesia^a^ (25,886)		
	%		%		
Age (years)					<0.001
18-54	16.87		14.10		
55-64	28.47		28.39		
65-74	29.51		32.97		
75 and older	25.15		24.54		
BMI					<0.001
Underweight	1.72		1.00		
Normal	21.48		21.91		
Overweight	31.31		33.99		
Class I	24.71		25.16		
Class II	13.37		12.35		
Class III	7.40		5.60		
Gender					0.020
Female	56.59		55.65		
Male	43.41		44.35		
Current Smoker within one year					<0.001
No	85.21		88.50		
Yes	14.79		11.50		
Diabetes mellitus with oral agents or insulin					<0.001
Insulin	3.52		2.53		
Non-insulin	9.86		8.30		
No	86.61		89.18		
Hypertension requiring medication					<0.001
No	40.90		47.67		
Yes	59.10		52.33		
Disseminated cancer					<0.001
No	99.17		99.73		
Yes	0.83		0.27		
Steroid use for a chronic condition					<0.001
No	95.86		96.65		
Yes	4.14		3.35		
Functional health status prior to surgery					<0.001
Independent	95.77		97.66		
Partially Dependent	3.70		2.12		
Totally Dependent	0.52		0.21		
Elective Surgery					<0.001
No	14.24		5.97		
Yes	85.76		94.03		
ASA Classification					<0.001
No/Mild Disturb^b^	48.86		59.89		
Severe Systemic Disease	51.14		40.11		

Table 2 describes the unadjusted frequency distribution of 30 days postoperative all causes mortality. When analyzing THA and PHA patients together in one group, patients who received general anesthesia had significantly higher early postoperative mortality than patients who received regional anesthesia (0.77% vs. 0.33%, p < 0.001). Except for gender, there were significant differences in mortality according to all examined control variables. Early postoperative mortality was higher in the following patient groups: individuals aged 75 or older, individuals with an underweight BMI (less than 18.5), non-smokers with multiple comorbidities, individuals taking corticosteroids, those with non-elective/emergency surgery, individuals with low preoperative functional status scores, and those with ASA III or higher classification.

**Table 2:** Characteristics of Hip Arthroplasty Patients at Risk for Postoperative Mortality (N = 60,897) ^a^“Regional anesthesia” is defined as patients who received intraoperative regional, epidural, or spinal anesthesia, as per the ACS NSQIP database. ^b^“No/mild disturb” is defined as patients under the ASA classification (1) no disturb or (2) mild disturb. BMI: body mass index; ACS NSQIP: American College of Surgeons National Surgical Quality Improvement Program; ASA: American Society of Anesthesiologists

Patient Characteristics	Postoperative Mortality (Death within 30 days of operation)				P-Value
	Alive		Dead		
	%		%		
Types of Anesthesia					<0.001
General Anesthesia	99.23		0.77		
Regional Anesthesia^a ^	99.67		0.33		
Age (years)					<0.001
18-54	99.87		0.13		
55-64	99.87		0.13		
65-74	99.78		0.22		
75 and older	98.17		1.83		
BMI					<0.001
Underweight	95.18		4.82		
Normal	98.93		1.07		
Overweight	99.61		0.39		
Class I	99.81		0.19		
Class II	99.73		0.27		
Class III	99.80		0.20		
Gender					0.174
Female	99.38		0.62		
Male	99.47		0.53		
Current Smoker within one year					0.037
No	99.40		0.60		
Yes	99.58		0.42		
Diabetes mellitus with oral agents or insulin					<0.001
Insulin	98.73		1.27		
Non-insulin	99.32		0.68		
No	99.46		0.54		
Hypertension requiring medication					<0.001
No	99.61		0.39		
Yes	99.27		0.73		
Disseminated cancer					<0.001
No	99.46		0.54		
Yes	93.37		6.63		
Steroid use for a chronic condition					0.017
No	99.43		0.57		
Yes	99.05		0.95		
Functional health status prior to surgery					<0.001
Independent	99.61		0.39		
Partially Dependent	95.06		4.94		
Totally Dependent	86.92		13.08		
Elective Surgery					<0.001
No	96.02		3.98		
Yes	99.83		0.17		
ASA Classification					<0.001
No/Mild Disturb^b ^	99.90		0.10		
Severe Systemic Disease	98.86		1.14		

However, when separately analyzing patients who underwent THA or PHA, as reported in Table 3, there was no significant association between the type of anesthesia administered perioperatively and early postoperative mortality in either group. The adjusted odds ratio of early postoperative mortality in patients undergoing PHA was not significant when comparing regional anesthesia to general anesthesia (odds ratio {OR} = 0.85; confidence interval {CI} 0.59-1.22; p = 0.39). Even after adjustment, there were no significant associations between the type of anesthesia and postoperative mortality in either PHA or THA patients (p = 0.10).

**Table 3:** Interaction Assessment of Hip Arthroplasty Type ^a^Adjusted by age, BMI, sex, diabetes status, hypertension, disseminated cancer, steroid use, functional status, elective surgery, and ASA classification. PHA: partial hip arthroplasty; THA: total hip arthroplasty; BMI: body mass index; ASA: American Society of Anesthesiologists

	Unadjusted				Adjusted^a^			
	PHA		THA		PHA		THA	
	OR (95% CI)	P-value	OR (95% CI)	P-value	OR (95% CI)	P-value	OR (95% CI)	P-value
Type of Anesthesia								
General	Reference		Reference		Reference		Reference	
Regional	0.83 (0.61 - 1.13)	0.23	0.47 (0.31 - 0.73)	<0.001	0.85 (0.59 - 1.22)	0.39	0.68 (0.43 - 1.08)	0.100

## Discussion

Around the world, the number of hip arthroplasties performed has risen significantly due to its wide range of indications such as osteoarthritis and hip fractures [19]. To facilitate optimal outcomes for patients, it is important to understand the complications associated with hip arthroplasty and the factors that contribute to those complications [20]. In this study, we assessed early postoperative mortality following THA or PHA, given the type of anesthesia administered perioperatively. After adjusting for potential confounders, we found there was no significant difference in early postoperative mortality in THA or PHA patients when general or regional anesthesia was administered. It was important for us to address any confounding by indication. In other words, we needed to consider the possibility that the higher early postoperative mortality rate observed in the general anesthesia group may have resulted from a greater propensity for life-threatening comorbidities associated with said group. Having assessed the role of confounders in our study, we affirm that the results are likely to be a sound and unbiased estimation of the association between the type of anesthesia administered and postoperative mortality in THA and PHA patients.

Our results concur with the results of two previously published studies. The retrospective systematic review of randomized control trials by Macfarlane et al. yielded two trials that documented mortality as a secondary outcome to total hip arthroplasty and neither produced significant results [18]. Seven years later, Johnson et al. performed a retrospective systematic review that looked more promising with seven of its trials documenting mortality as a secondary outcome, yet none of them yielded significant results [15]. These reports may not rule out a difference in mortality according to the type of anesthesia since mortality after hip arthroplasty is an infrequent outcome; therefore, these studies were likely underpowered. One study in the literature found results that may conflict with our findings: the Basques et al. study [13]. It also utilized the ACS NSQIP database and studied a similar outcome of interest; however, the outcome of interest was more general; the 30-day risk of an adverse event. While this study found that general anesthesia was significantly associated with an increase in the 30-day risk of an adverse event, it is important to note that this study did not explore the 30-day risk of postoperative mortality [13].

While our study found that there is no difference in early postoperative mortality between patients undergoing THA or PHA and receiving general or regional anesthesia, other studies have found there may be differences in other postoperative complications and outcomes. It has been reported that patients undergoing ambulatory orthopedic procedures under general anesthesia had a faster time to initial micturition and unassisted ambulation when compared with spinal anesthesia, which can be significant in our patient population that is undergoing hip surgery [21]. While the use of general anesthesia in patients undergoing THA has been associated with increased postoperative adverse complications such as stroke and cardiac arrest, it was not associated with longer postoperative hospital length of stay or 30-day readmissions, which suggests that patients may be at an immediately increased risk of adverse outcomes postoperatively, but that risk decreases rapidly over time, likely as the body recovers from the effects of the general anesthetic agent [13,22]. It is important to consider all of the potential postoperative complications and outcomes when counseling patients and making decisions on what form of anesthesia to utilize in patients undergoing THA or PHA. 

There are some limitations of our study, including the grouping of the different subtypes of regional anesthesia into one category, which may affect the internal validity of our study because we cannot specifically identify whether there were differences in outcomes between the different regional anesthesia techniques. Larger sample size would be necessary in order to assess the individual effects of different respective subgroups of regional anesthesia. Additionally, there is a lack of specification of which anesthesia medications were administered perioperatively, and no mention of the delivery technique or dosage of the anesthetic medication, which can influence perioperative complications. Finally, patients were analyzed by the procedure they underwent, rather than by the injury or condition they were being treated for, which could influence postoperative mortality. Further study should be performed to assess these variables.

## Conclusions

The findings of this study indicate that total hip arthroplasty and partial hip arthroplasty are safe procedures with low early postoperative mortality and that utilizing general anesthesia in adult patients undergoing either a THA or PHA is not an independent risk factor for early postoperative mortality. Considering the multitude of factors that determine the type of anesthesia that is given during total or partial hip arthroplasty, the results of this study indicate that the use of regional anesthesia may not be protective for early postoperative mortality in these patients.
